# Proteostasis Regulation in the Endoplasmic Reticulum: An Emerging Theme in the Molecular Pathology and Therapeutic Management of Familial Hypercholesterolemia

**DOI:** 10.3389/fgene.2020.570355

**Published:** 2020-09-23

**Authors:** Deepu Oommen, Praseetha Kizhakkedath, Aseel A. Jawabri, Divya Saro Varghese, Bassam R. Ali

**Affiliations:** ^1^Department of Genetics and Genomics, College of Medicine and Health Sciences, United Arab Emirates University, Al-Ain, United Arab Emirates; ^2^Department of Pathology, College of Medicine and Health Sciences, United Arab Emirates University, Al-Ain, United Arab Emirates; ^3^Zayed Center for Health Sciences, College of Medicine and Health Sciences, United Arab Emirates University, Al-Ain, United Arab Emirates

**Keywords:** ERAD pathway, cholesterol, familial hypercholesterolemia, Class II mutations, lipid metabolism, LDLR, ER stress, unfolded protein response

## Abstract

Familial hypercholesterolemia (FH) is an autosomal genetic disease characterized by high serum low-density lipoprotein (LDL) content leading to premature coronary artery disease. The main genetic and molecular causes of FH are mutations in low-density lipoprotein receptor gene (*LDLR*) resulting in the non-clearance of LDL from the blood by hepatocytes and consequently the formation of plaques. LDLR is synthesized and glycosylated in the endoplasmic reticulum (ER) and then transported to the plasma membrane via Golgi. It is estimated that more than 50% of reported FH-causing mutations in LDLR result in misfolded proteins that are transport-defective and hence retained in ER. ER accumulation of misfolded proteins causes ER-stress and activates unfolded protein response (UPR). UPR aids protein folding, blocks further protein synthesis, and eliminates misfolded proteins via ER-associated degradation (ERAD) to alleviate ER stress. Various studies demonstrated that ER-retained LDLR mutants are subjected to ERAD. Interestingly, chemical chaperones and genetic or pharmacological inhibition of ERAD have been reported to rescue the transport defective mutant LDLR alleles from ERAD and restore their ER-Golgi transport resulting in the expression of functional plasma membrane LDLR. This suggests the possibility of pharmacological modulation of proteostasis in the ER as a therapeutic strategy for FH. In this review, we picture a detailed analysis of UPR and the ERAD processes activated by ER-retained LDLR mutants associated with FH. In addition, we discuss and critically evaluate the potential role of chemical chaperones and ERAD modulators in the therapeutic management of FH.

## Introduction

### Familial Hypercholesterolemia

Familial hypercholesterolemia (FH) is a genetic disorder that results in altered lipid metabolism and consequently leading to elevated levels of plasma low-density lipoprotein cholesterol (LDL-C) (Soutar and Naoumova, 2007). Clinically, FH is characterized by increased levels of LDL-C, tendon xanthomas, corneal arcus, and premature coronary artery diseases (CAD) such as atherosclerosis (Müller, 1938; Kawaguchi et al., 1999; Kitahara et al., 2019). Mutations in the low-density lipoprotein receptor gene (*LDLR*) account for more than 80% of monogenic FH (Brown et al., 1986) (FHCL1, OMIM#143890). Monogenic FH can also be caused by mutations in other genes including *APOB* (FHCL2, OMIM#144010) (Innerarity et al., 1990), *PCSK9* (FHCL3, OMIM#603776) (Abifadel et al., 2003), and *LDLRAP1* (FHCL4, OMIM# 603813) (Garcia et al., 2001). FH can exist in both heterozygous and homozygous forms with homozygous FH (HoFH) patients at far greater risk of developing CAD in their first decade of life (Alonso et al., 2014). The clinical manifestations of a homozygous patient suffering from FH begin in the first decade of their life including abnormal cholesterol storage which results in the appearance of cutaneous xanthomas and the appearance of tendon xanthomas particularly in the joints and fingers. Another late symptom is the manifestation of xanthelasmata as well as corneal arcus. Also, coronary manifestations in HoFH appear in their second and third decades (Klose et al., 2014) though fatal myocardial infarctions (MIs) are possible even in early childhood (Wiegman et al., 2015). On the other hand, the clinical manifestations in heterozygous FH patients are possible from early adulthood onward and premature CAD in the second or third decade of life. Sometimes symptoms may remain clinically hidden (Klose et al., 2014). If left untreated, approximately 50% heterozygous males and 15% females have a fatal MI by the age of 60 (Henderson et al., 2016). In recent studies, it has been shown that the prevalence of heterozygous FH has increased and affects between 1:200 or 1:300 in most populations (Nordestgaard et al., 2013).

Cholesterol is an essential component of membranes and serves as a precursor for steroid molecules such as hormones, bile acids and vitamin D. Cellular cholesterol requirement is met either by *de novo* intracellular synthesis or by uptake of dietary cholesterol (Goldstein and Brown, 1990). Receptor-mediated endocytosis of cholesterol mediated by LDLR, unraveled by the seminal work of Brown and Goldstein, is the main pathway for cellular uptake of exogenous cholesterol (Brown et al., 1986). On the cell membrane, the LDLR receptors are localized to clathrin-coated pits and when the LDL-bound cholesterol attaches to the receptor, the complex is internalized and fuse with early sorting endosomes. There the receptor dissociates from the lipid and recycles back to the cell-surface repeating this cycle every 10 min (Brown et al., 1986). The LDL particles are eventually delivered via endosomal trafficking to the lysosomes for degradation and the cholesterol is released within the cell. Excess cellular cholesterol is esterified and stored in lipid droplets in the endoplasmic reticulum (ER) (Ikonen, 2008). Cellular cholesterol homeostasis is a tightly regulated process and the ER plays a crucial role in cholesterol sensing, regulation, and synthesis (Röhrl and Stangl, 2018). The ER is also the site of synthesis of many membrane proteins including that of LDLR which is in turn subject to feedback regulation by intracellular cholesterol levels. The review aims to present how LDLR mutants implicated in FH deregulates ER homeostasis and also explores the possibilities of targeting ER-proteostasis machinery for therapeutic management of FH.

### Low-Density Lipoprotein Receptor (LDLR): Gene, Protein Structure, and Function

**FIGURE 1:**
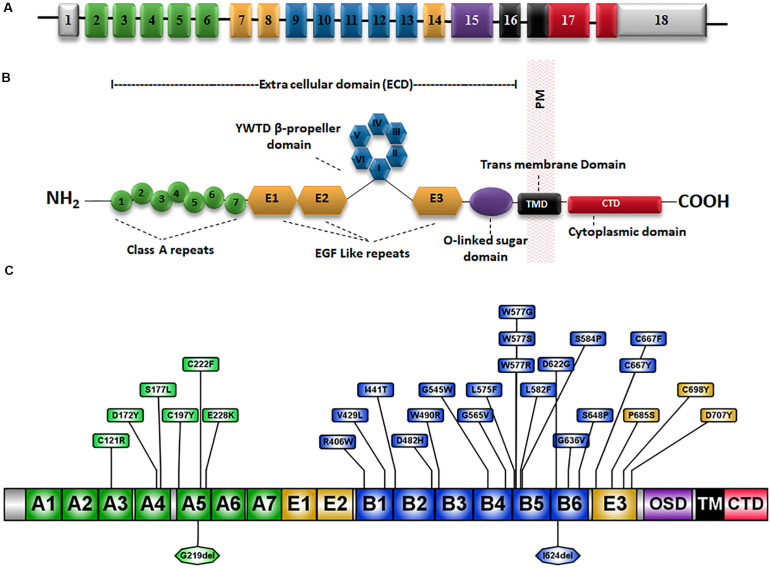
Schematic representation of the *LDLR* gene and protein structure. **(A)** The 18 exons of *LDLR* are numbered and exons coding for different domains of the LDLR protein are represented by different colors. **(B)** The LDLR protein has an extracellular domain (ECD), a membrane-spanning domain (TMD) and a cytoplasmic *C*-terminal domain. The ECD consists of a ligand-binding domain (LBD), an epidermal growth factor (EGF) homology domain and an *O*-linked sugar region. The EGF homology domain is composed of three EGF-like repeats 1–3 and a β-propeller domain of six YWTD motifs occurs between repeats 2 and 3. **(C)** Schematic diagram showing the amino acid positions of well known class II mutations (Table 2) and the substitutions are marked in boxes. OSD, *O*-linked sugar domain.

The low-density lipoprotein receptor (LDLR) is the prototype receptor of a group of structurally and functionally similar cell surface receptors. LDLR is encoded by the *LDLR* gene located on chromosome 19p13.1-13.3. It spans ∼45 kb and comprises 18 exons that are translated into 860 amino acids including a signal sequence of 21 amino acids which is cleaved during translocation into the ER (Francke et al., 1984) (Figure 1A). Each exon or group of exons constitutes a particular domain in the LDLR (Figure 1A) (Gent and Braakman, 2004). There are five LDLR domains and each domain mediates a specific function (Klee and Zimmermann, 2019) which are: a ligand-binding domain (LBD), an epidermal growth factor (EGF) homology domain, an *O*-linked sugar region, a membrane-spanning domain and a *C*-terminal cytoplasmic tail domain (Gent and Braakman, 2004) (Figure 1B).

The LBD is made up of 292 amino acids and consists of seven cysteine-rich ligand-binding repeats (LRs) each composed of 40 amino acid residues (Yamamoto et al., 1984; Südhof et al., 1985; Fass et al., 1997). Six cysteine residues along with a group of negatively charged amino acids in the LR sequence interact with positively charged residues on the APOB and APOE molecules to mediate the recognition and binding of LDL-C to LDLR (Bradley and Gianturco, 1986). The second domain is the EGF precursor domain which is composed of 400 amino acids and contributes to the dissociation of the LDL particles from the LDLR-LDL complex in the endosome at a low pH (Davis et al., 1987; Rudenko et al., 2002). The EGF precursor domain is composed of three EGF-like repeats, EGF-like 1, 2, and 3 each consisting of ∼40 amino acids. A domain of six YWTD motifs known as β-propeller domain occurs between repeats 2 and 3 (Springer, 1998; Jeon et al., 2001) (Figure 1B).

The *O*-linked sugars domain plays a major role in the post-translational modification of LDLR. This domain is encoded by exon 15 and is composed of 48 amino acids consisting of 18 threonine and cysteine residues that act as attachment sites for *O*-linked sugar chains. The membrane-spanning domain is responsible for LDLR integration and attachment to the cell membrane (Russell et al., 1984; Yamamoto et al., 1984; Südhof et al., 1985). Endocytosis of LDLR-LDL complex is mediated by the 5th domain in the LDLR which is the cytoplasmic tail encoded by exon 17 and exon 18 (Goldstein and Brown, 2009).

### LDLR Mutations-Types and Classifications

A total of 2,299 variants have been reported in association with FH in the Human Gene Mutation Database (HGMD) (Stenson et al., 2017), ranging between missense mutations, nonsense mutations, deletions, insertions or duplications. Based on functional consequences, LDLR mutations have been classified into five major classes (Hobbs et al., 1992), as described in detail in Table 1. Briefly, the functional impact of each classes are, Class I: Defects in synthesis of LDLR mainly due to null alleles; Class II: Impaired trafficking of the LDLR to Golgi compartments and cell surface, due to improper folding and complete or partial retention in the ER (2A and 2B, respectively); Class III: Deficient in binding to ligands; Class IV: Impaired clustering and endocytosis of ligand-bound receptors; Class V: Interferes with the cell-surface recycling of internalized LDLR due to defects in dissociation of ligand from the receptor, subsequently leading to the degradation of the receptor in the lysosome (Beglova et al., 2004; Van Hoof et al., 2005). Class VI is a new functional class of LDLR variants where the LDLR is properly synthesized by the ER and Golgi apparatus but fail to undergo basolateral sorting in polarized cells (Koivisto et al., 2001). Additional novel functional classes are emerging with increasing functional data (Susan-Resiga et al., 2017) and most recently a novel class of LDLR variants inducing ectodomain cleavage of the LDL receptor in the ER has been suggested (Strøm et al., 2014, 2017).

**TABLE 1:** Classes of LDLR variants.

LDLR variant classes	Type of variants	Protein/functional impact
Class I	• Early stop codons • Mutations in the promoter regions • Splicing aberrations • Large exonic deletions	Synthesis defective: Defects in LDLR protein synthesis

Class II: • Class II A • Class II B	• Missense mutations in the cysteine-rich domains • In-frame deletions/duplications • Protein truncating mutations	Transport defective: Defects in LDLR folding, maturation and transport in the secretory pathway Class IIA: Completely retained in the ER due to folding defects Class II B: Transport-competent but ER-retained due to slower processing

Class III	• Point mutations clustering in the ligand binding domain	Binding defective: Transport-competent but defective in binding to LDL

Class IV	• Mutations in the 4th and 5th domains • Complete deletion of those LDLR domains	Clustering and endocytosis defective: Impair with the clustering of ligand-bound LDLR in clathrin coated pits and endocytosis of LDLR-LDL complex

Class V	• Deletions in the EGF precursor domain	Dissociation and recycling defective: The LDLR-LDL complex is successfully internalized in the cell, but dissociation of the LDLR from the LDL does not happen leading to the degradation of LDLR along with LDL in the lysosomes (Beglova et al., 2004; Van Hoof et al., 2005)

Around 50% of reported LDLR mutations are Class II mutants which are implicated to be transport-defective (Varret and Rabès, 2012). At present, there are 895 missense mutations reported in the HGMD (Stenson et al., 2017), occurring at 451 amino acid codons distributed across the whole length of the protein (Supplementary Figure S1). However, only limited information is available on the functional classes of these variants (Benito-Vicente et al., 2018). A compilation of functionally validated Class II mutations from the published literature is presented in Table 2. A schematic representation of the position of occurrence of the reported variants is shown in Figure 1C. Unlike the other classes of mutants that interfere with a specific function of the receptor, class II mutations cause global conformation defects leading to their retention in the ER, potentially overwhelming the cellular proteostasis machinery in addition to impaired cholesterol homeostasis (Gent and Braakman, 2004).

**TABLE 2:** List of all functionally characterized Class II LDLR variants.

LDLR (NM_000527.4; NP_000518.1) class II variants with functional evidence	Variant class	References	Population frequency (gnomAD)	dbSNP ID
c.361T > C(p.C121R)	Class II	Guo et al. (2019)	N/A	rs879254492
c.514G > T(p.D172Y)	Class IIB	Jeenduang et al. (2010)	N/A	rs879254554
c.530C > T(p.S177L)	Class IIB	Li et al. (2004)	1.59E-05	rs121908026
c.590G > A(p.C197Y)	Class IIB	Li et al. (2004)	3.19E-05	rs376459828
c.665G > T(p.C222F)	Class IIB	Wang et al. (2014)	N/A	rs730882086
c.682G > A(p.E228K)	Class IIA	Li et al. (2004)	1.61E-05	rs121908029
c.1216C > T(p.R406W)	Class IIB or V	Benito-Vicente et al. (2015)	1.77E-05	rs121908043
c.1285G > C(p.V429L)	Class IIA	Etxebarria et al. (2014)	N/A	rs28942078
c.1322T > C(p.I441T)	Class IIA	Benito-Vicente et al. (2015)	N/A	rs879254862
c.1444G > C(p.D482H)	Class II	Kizhakkedath et al. (2019)	N/A	rs139624145
c.1468T > C(p.W490R)	Class IIA	Etxebarria et al. (2014)	N/A	rs730880130
c.1633G > T(p.G545W)	Class IIA	Benito-Vicente et al. (2015)	N/A	rs879254965
c.1694G > T(p.G565V)	Class II	Esser and Russell (1988)	N/A	rs28942082
c.1723C > T(p.L575F)	Class II	Jiang et al. (2016)	3.98E-06	rs1205480064
c.1729T > G(p.W577G)	Class IIA	Etxebarria et al. (2015)	N/A	rs879255000
c.1729T > C(p.W577R)	Class II	Schaefer et al. (2012)	N/A	rs879255000
c.1730G > C(p.W577S)	Class II	Holst et al. (2001)	7.95E-06	rs138947766
c.1744C > T(p.L582F)	Class II	Jiang et al. (2016)	N/A	rs1131692216
c.1750T > C(p.Ser584Pro)	Class IIA	Galicia-Garcia et al. (2020)	N/A	rs879255010
c.1775G > A(p.Gly592Glu)	Class IIB	Susan-Resiga et al. (2017)	5.66E-05	rs137929307
c.1865A > G(p.Asp622Gly)	Class IIA	Galicia-Garcia et al. (2020)	N/A	rs879255060
c.1907G > T(p.G636V)	Class IIB	Wang et al. (2014)	N/A	N/A
c.1942T > C(p.S648P)	Class IIB	Etxebarria et al. (2014)	N/A	rs879255079
c.2000G > T(p.C667F)	Class II	Kizhakkedath et al. (2019)	N/A	rs28942083
c.2000G > A(p.C667Y)	Class IIA	Li et al. (2004)	3.98E-06	rs28942083
c.2053C > T(p.P685S)	Class IIB	Etxebarria et al. (2014)	N/A	rs2569548
c.2093 G > A(p.Cys698Tyr)	Class IIA	Galicia-Garcia et al. (2020)	N/A	rs879255136
c.2119 G > T (p.Asp707Tyr)	Class IIA	Galicia-Garcia et al. (2020)	N/A	rs879255142
c.654_656delTGG (p.Gly219del)^a^	Class II	Omer et al. (2017)	2.79E-05	rs121908027
c.1871_1873delTCA (p.Ile624del)^a^	Class II	Etxebarria et al. (2015)	N/A	rs879255062
c.1878delA (p.Ala627Profs*38)^b^	Class II	Banerjee et al. (2019)	N/A	rs1057516134
c.2043C > A (p.Cys681Ter)^b^	Class II	Banerjee et al. (2019)	7.96E-06	rs121908031
c.2399_2403delTCTTCinsGGGT (p.Val800Glyfs*129)^b^	Class II	Etxebarria et al. (2015)	N/A	rs879255198
c.1885_1889delTTCAGinsGATCATCAACC (p.Phe629_Ser630delinsAspHisGlnPro)^c^	Class II	Shu et al. (2017)	N/A	N/A

*^a^In-frame amino acid deletions. ^b^Protein truncating variant. ^c^Complex deletion-insertion.*

## Mechanisms of Protein Quality Control and Proteostasis Regulation in the ER

In eukaryotes, an estimated one-third of all newly synthesized proteins enter the ER to undergo post-translational modifications and achieve their three-dimensional native conformation, before reaching their proper cellular destination (Brodsky and Skach, 2011). However, protein folding is an inherently error-prone process and only a fraction of all produced proteins reaches a native conformation. Multiple stringent quality control mechanisms operates in the ER to ensure that only properly folded proteins are transported out of the ER and protein homeostasis or “proteostasis” is maintained (Sun and Brodsky, 2019). Many membrane and secretory proteins that fail to conform to the ER quality control (ERQC) are dislocated into the cytosol and degraded by the proteasome by a process termed as ER-associated degradation (ERAD) (Vembar and Brodsky, 2008; Ruggiano et al., 2014; Sun and Brodsky, 2019). Misfolded proteins can still retain their function and premature ERAD of mutant misfolded proteins is accounted for the cellular pathogenesis of several congenital disorders (Ward et al., 1995; Hume et al., 2009; Ali et al., 2011; Al-Kindi et al., 2014; Kizhakkedath et al., 2014, 2019; John et al., 2015). Sometimes the quality control mechanisms fail to recognize folding-incompetent forms which leads to the accumulation of folding-intermediates in the ER, causing ER stress. The cells respond to ER stress by initiating the unfolded protein response (UPR), an integrated stress response program, that aims to increase cell’s folding capacity, accelerate clearance of unfolded proteins by ERAD, and restore protein homeostasis in the cell (Karagöz et al., 2019). Unresolved ER stress may lead to cell death (Karagöz et al., 2019). The ER-retained LDLR class II mutants have been reported to be degraded through a proteasome-mediated pathway (Li et al., 2004) and have been shown to activate ER-stress pathways (Sørensen et al., 2006).

### Major Components of ERAD

ER-associated degradation is a collective term for a succession of events that starts with substrate recognition, followed by chaperone-assisted translocation to the cytosol and culminates in degradation by the ubiquitin-proteasome system (UPS) (Brodsky and Skach, 2011; Sun and Brodsky, 2019). Though complex, the fundamental ERAD machinery is conserved in eukaryotes from yeast to mammals (Brodsky and Skach, 2011; Sun and Brodsky, 2019). The folding of nascent polypeptides entering the ER is assisted by a chaperone system comprising of classical ER chaperones, lectin chaperones and protein disulfide isomerases (PDIs) (Braakman and Hebert, 2013). Classical chaperones belonging to the heat shock proteins (HSPs) family are GRP78/BiP (Hsp70), GRP94 (Hsp90), and J-proteins (Hsp40) (Braakman and Hebert, 2013). GRP78 recognizes and binds to misfolded proteins with exposed hydrophobic residues and helps in interaction other HSP chaperones and PDIs (Ni and Lee, 2007). *N*-linked glycosylation of Asn-X-Ser/Thr motif is an important post-translational modification that help nascent proteins to remain soluble and prevent aggregation by masking the hydrophobic stretches in the protein (Aebi et al., 2010). *N*-glycosylation involves the attachment of a preassembled carbohydrate, comprised of three glucoses, nine mannoses, and two *N*-acetyl glucosamines (Glc_3_Man_9_GlcNAc_2_), to the Asn residue (Aebi et al., 2010).

**FIGURE 2:**
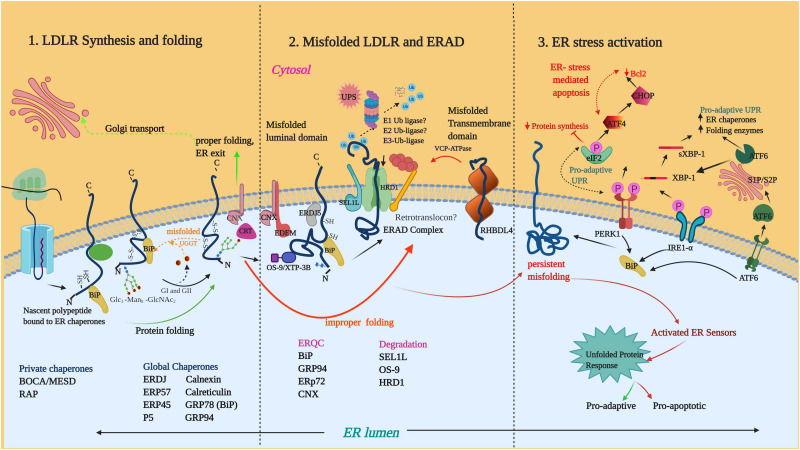
LDLR folding, misfolding and activation of UPR: **(1)** The nascent LDLR is co-translationally inserted into the ER membrane and the LDLR ectodomain undergoes folding in the ER lumen with the assistance of several global and private chaperones as listed in the figure. **(2)** Misfolded proteins such as Class II mutants engage in prolonged interaction with the chaperone system. BiP, GRP94, ERP72 are ERQC factors implicated in LDLR retention. Terminally misfolded proteins are extracted from the chaperone system and delivered to membrane-embedded ERAD complex for degradation by the ubiquitin-proteasome system. So far, the components known to be involved in LDLR-ERAD are OS9, SEL1L and HRD1. RHBDL4 is a metalloprotease involved in the ERQC of ERAD-M candidates of LDLR. **(3)** Accumulation of misfolded LDLR induces ER stress and activates the UPR proteins IRE1, PERK, and ATF6. Phosphorylation of eIF2α by PERK leads to the attenuation of protein translation. Activated IRE1α induces splicing of the long XBP1 mRNA to form XBP1s mRNA which encodes XBP1s protein. Activated ATF6 is cleaved in the Golgi to form the active ATF6 N-terminal fragment. XBP1s and ATF6 are transcription factors that target the transcriptional induction of UPR target genes. Unresolved ER-stress turn-on proapoptotic pathways through the PERK-arm of the UPR. Illustration created with Biorender.com.

Enzymatic deglucosylation of the *N*-glycan to Glc_1_Man_9_GlcNAc_2_ by glucosidases I (GI) and 2 (GII), makes it a high-affinity ligand for lectin chaperones such as calnexin (CNX) and calreticulin (CRT). Binding of CNX/CRT to glycoproteins facilitates their retention in ER, prevention of aggregation and recruitment of PDIs such as ERp57 (PDIA3) (Zapun et al., 1998; Lamriben et al., 2016). Removal of the final glucose by GII prevents further binding CNX/CRT and if folded, the substrates progress toward ER exit sites. Unfolded proteins undergo further rounds of reglucosylation by UDP-glucose/glycoprotein glucosyltransferase (UGGT) and are reverted to CNX/CRT for folding. If folded the glycoproteins eventually exit the cycle (Lamriben et al., 2016) and terminally misfolded proteins are released from this cycle and diverted to ERAD for disposal. An example of glycoprotein folding is that of LDLR which is represented in Figure 2.

Demannosylation by the ER-resident mannosidases such as ER mannosidase 1 (ERMan1) and ER-degradation enhancing mannosidase-like proteins (EDEM1, 2, and 3) results in *N*-glycans with deglucosylated, demannosylated forms (Man5–Man7) that are incompatible with UGGT-mediated reglucosylation (Shenkman and Lederkremer, 2019). The deglycosylated and demannosylated misfolded proteins are selectively captured by the mannose-specific lectins (OS9 and XTP-3B) for their further delivery to the ER degradation machinery (Olzmann et al., 2013) (Figure 2). Non-glycosylated misfolded proteins are also targeted for ERAD and it is believed that features other than glycan trimming may contribute to their recognition (Okuda-Shimizu and Hendershot, 2007). Non-glycosylated proteins can be directly recruited by BiP (GRP78) and J-domain proteins to ERAD complex, bypassing the CNX pathway (Ushioda et al., 2013).

Once selected, the ERAD substrates are delivered to the cytosol for ubiquitination by E3-ubiquitin ligases and proteolytic degradation by the UPS. In yeast, specialized ERAD pathways exist to degrade misfolded proteins with defects exposed in the ER luminal (ERAD-L), transmembrane (ERAD-M), and cytosolic domains (ERAD-C) (Carvalho et al., 2006). An emerging body of evidence suggests that mammalian ERAD does not follow rigid rules for substrate engagement and an array of E3 ligases cooperates to complete the ERAD processing of substrates with diverse topologies (Christianson et al., 2011; Olzmann et al., 2013). Two well-known mammalian E3 ubiquitin ligases are polytopic RING domain ubiquitin ligases, HMG-CoA reductase degradation protein 1 (HRD1/SYVN1) and gp78/autocrine motility factor receptor (AMFR) (Fang et al., 2001; Nadav et al., 2003). E3 ligases such as RMA1 (RNF5), TRC8, TEB4 (MARCH IV) have been reported to be involved in the degradation of a limited number of ERAD clients (Olzmann et al., 2013).

Since the proteins targeted for degradation have diverse structures and topologies, distinct combinations of adaptors that recognize these features are recruited by the E3-ubiquitin ligases. In mammals, the transmembrane (TM) protein SEL1L works in conjunction with the HRD1 E3 ligase and is necessary to deliver the ERAD substrates from ER lectins (OS9, XTP) to HRD1 (Christianson et al., 2008; Hosokawa et al., 2008). Depletion of SEL1L has been reported to destabilize HRD1 and prevent the degradation of misfolded lumenal/TM proteins (Christianson et al., 2008; Horimoto et al., 2013; Bianchini et al., 2014; Kizhakkedath et al., 2018). Other mammalian ER adapters are ERLINSs, INSIGs and F-box proteins (Olzmann et al., 2013).

Derlin family of proteins DER1, 2, and 3 have been proposed to play a role in substrate dislocation through association with HRD1/SEL1L (Lilley and Ploegh, 2005). During dislocation, the disulfide bonds are reduced by oxidoreductase enzymes like ERFAD and ERDJ5 (Smith et al., 2011) and partially unfolded by rhomboid pseudoproteases such as Derlins, UBCA2 and UBXD8 (Olzmann et al., 2013). The dislocation process is powered by the cytosolic valosin containing protein (VCP)/p97 ATPase (Guerriero and Brodsky, 2012). During dislocation, an E1 ubiquitin ligase enzyme activates ubiquitin and an ubiquitin-conjugating enzyme (E2 ligase) in conjunction with a ubiquitin ligase (E3) then transfer ubiquitin to the substrate (Christianson and Ye, 2014). UBA1 is a well characterized E1 ligase enzyme in humans and initially believed to be the only subtype of E1 ligases. Examples of mammalian E2 ligases are UBE2J1, UBE2J2 and UBE2G2. The ubiquitin-tagged substrates are then delivered to degradation by the 26S proteasome in the cytosol with or without the help of small cytosolic heat-shock chaperones (Christianson and Ye, 2014) (Figure 2).

### Quality Control of Membrane Proteins With Defective Transmembrane Domains

The quality control of TM proteins containing defects in their membrane-spanning domain appear to be distinct from that of ERAD-L and ERAD-C substrates, since luminal or cytosolic factors do not have access to the domain location. An intramembrane rhomboid protease, RHBDL4 has been shown to cleave TM-anchors of unstable single-membrane spanning or polytopic membrane proteins in an ubiquitin-dependent manner and divert them to the UPS coupled to VCP/p97 ATPase (Figure 2) (Fleig et al., 2012). Recent studies have shown that the ERAD-M substrates containing less hydrophobic TM-domains get translocated entirely to the ER lumen leading to recognition by BiP and degradation through the canonical ERAD-L pathway (Feige and Hendershot, 2013). Another study proposed that degradation of an ERAD-M substrate containing an unassembled TMD was dependent upon ubiquitination on cytoplasmic lysine residues and occurs through a specific ERAD pathway that is mechanistically distinct from that which mediates degradation of membrane proteins with luminal folding defects (Briant et al., 2015).

### Non-canonical ERAD Pathways

While ERAD is highly efficient in handling a variety of misfolded proteins, some membrane and soluble proteins form aggregates and place constraints on ER retrotranslocation machinery. These aggregates are diverted to the lysosome for degradation via alternative pathways collectively termed as ER-to-lysosome–associated degradation (ERLAD) (Houck et al., 2014; Fregno and Molinari, 2019; De Leonibus et al., 2019). ERLAD include (i) ER-phagy, (ii) microautophagy, and (iii) vesicular transport (De Leonibus et al., 2019; Fregno and Molinari, 2019). In ER-phagy, ER fragments are engulfed by a double membrane LC3-decorated autophagosome that fuses with the lysosome. Microautophagy is an ER autophagy where misfolded proteins segregated on ER exit sites (ERES) coated with LC3 and COPII, are directly engulfed by lysosomal invagination or protrusion. Vesicular transport is mediated by single membrane ER-derived vesicles that bud from the ER and fuse with endolysosomes decorated with LC3 (De Leonibus et al., 2019). In ER-phagy, membrane-embedded LC3-binding receptors regulate the delivery of ER-subdomains to lysosome. In mammals the ER-phagy receptors include FAM134B, RTN3, SEC62, CCPG1, ATL3, and TEX264 (Khaminets et al., 2015).

## ERAD Processing of LDLR Mutants

### Canonical Role of ERAD in the Regulation of Sterol Synthesis

Other than performing as a quality control checkpoint, ERAD plays a quintessential role in providing protein quantity control as well in response to environmental demands (Hegde and Ploegh, 2010; Printsev et al., 2017). Cholesterol metabolism and homeostasis are tightly regulated processes and ubiquitin-dependent protein degradation is involved in transcriptional regulation, the synthesis, efflux and uptake of cholesterol (Sharpe et al., 2014). At the transcriptional level, cholesterol metabolism is regulated by the opposing actions of two transcription factors, namely sterol regulatory element-binding proteins (SREBPs) and the liver X receptors (LXRs) (Sharpe et al., 2014). Under low cellular cholesterol levels, SREBPs are involved in the transcriptional induction of genes required for *de novo* biosynthesis of cholesterol and LDLR for the uptake of cholesterol (Innerarity et al., 1990). Under elevated cellular cholesterol levels, LXRs induce genes involved in cholesterol efflux pathways and degradation of LDLR (Nadav et al., 2003).

The most widely known example of quantity control by ERAD is the post-translational feedback-regulation of 3-hydroxy-3-methyl-glutaryl-CoA reductase (HMGCR), a rate-limiting enzyme in the mevalonate pathway which produces cholesterol and other isoprenoids (DeBose-Boyd, 2008). The accumulation of sterols in ER membranes triggers the binding of HMGCR to ER-membrane proteins INSIG1 and INSIG2 which in turn recruit ubiquitin ligases GP78, TRC8, and RNF145 (Jo et al., 2011; Menzies et al., 2018). Ubiquitinated reductase is then extracted by VCP ATPase and delivered to the proteasome (Jo et al., 2011). Squalene monooxygenase/Epoxidase (SQLE) is another rate-limiting enzyme in the mevalonate pathway downstream of HMGCR and recent studies have shown that another ER-resident E3 ligase, MARCH6 is involved in the ubiquitin-proteasome degradation of SQLE (Loregger et al., 2015). MARCH6 is postulated to play a multifaceted role in cholesterol homeostasis as an endogenous negative modulator of SREBP and HMGCR (Loregger et al., 2015). The E3 ligases FBW7 and RNF20 are involved in the ubiquitin-dependent regulation of SREBPs (Sundqvist et al., 2005; Lee et al., 2014; Sharpe et al., 2014). Inhibition of cholesterol synthesis activates SREBP and transcriptional upregulation of LDLR.

Other than transcriptional regulation by SREBP, LDLR is post-translationally regulated by ubiquitin-dependent degradation mediated by the E3 ligase- inducible degrader of the LDLR (IDOL) (Zelcer et al., 2009). IDOL is transcriptionally controlled by LXRs and appear to preferentially ubiquitinate the cytoplasmic tails of plasma-membrane localized LDLR and mediate lysosomal rather than proteasomal degradation of the receptor (Zelcer et al., 2009). Interestingly, IDOL was found to be capable of regulating the ER located LDLR forms also, since a Class II LDLR mutant G546D was demonstrated to be degraded by IDOL by a lysosomal pathway (Zelcer et al., 2009).

### Molecular Players in the LDLR Folding Pathway

The LDLR receptor family has a modular organization consisting of LDL-repeats, EGF-like repeats with β-propeller, a single TM domain, and a small cytosolic tail (Figure 1B) (Gent and Braakman, 2004). Even though the different domains are organized from NH_2_-to-COOH terminus and the folding is co-translational, the nascent LDLR polypeptide is demonstrated to fold rapidly into compact structures by forming non-native disulfide bonds linking distant domains of the receptor (Jansens et al., 2002; Gent and Braakman, 2004). The non-native disulfides are later isomerized and native short-range disulfide bridges are formed with high efficiency and rarely lead to aggregate formation. The high-efficiency folding of LDLR requires the assistance of several general and private chaperones (Garcia et al., 2001). The HSP chaperone GRP78 and PDI family member ERDJ5 have been reported to be involved in the folding of LDLR (Gent and Braakman, 2004; Oka et al., 2013).

ERDJ5 (DNAJC10) is an ER-localized oxidoreductase containing J domain and thioredoxin domains important for its disulfide exchange activity (Oka et al., 2013). ERDJ5 is known to participate in the degradation pathway of misfolded proteins by reducing the disulfide bonds prior to retrotranslocation (Ushioda et al., 2008). ERDJ5 has been proposed to take part in the processing of non-native disulfide bonds in LDLR which is required for the native disulfide formation and proper folding (Oka et al., 2013). Another member of the PDI family of oxidoreductases, ERP57 is involved in the native disulfide bond formation of substrates in the ER and functions closely with both CNX and CRT (Jessop et al., 2007). ERP57 is indicated to be important for the isomerization of non-native disulfide bonds in LDLR (Jessop et al., 2007). Other PDI family members such as P5 and ERP45 are also reported to exhibit substrate specificity toward LDLR (Jessop et al., 2009).

In addition to the aforementioned chaperones, several private chaperones are involved in LDLR folding. The LBD of LDLR family members require the assistance of the receptor associated protein (RAP) for maturation, which prevents premature interaction of the domain with its ligands in the same compartment (Herz and Marschang, 2003). The BOCA/MESD family of chaperones is shown to be specifically required for the folding of the β-propeller domain that is contained within the EGF precursor homology region of LDLR (Culi et al., 2004). Calcium has been shown to an absolute requirement for LDLR folding in the ER and lack of calcium, even at very early folding stages, was reported to result in irreversible misfolding of the wild-type protein (Pena et al., 2010). A detailed depiction of LDLR folding is presented in Figure 2.

### Proteostasis Components Involved in the Degradation of LDLR Class II Mutants

It was demonstrated that different Class II mutants of LDLR affecting the LBD (S156L, C176Y, and E207K) and EGF domain (C646Y) were retained in the ER and degraded by a proteasome-dependent pathway in cell lines stably expressing the mutants (Li et al., 2004). One of the first ER factors discovered to be involved in the retention of mutant LDLR was the molecular chaperone GRP78/BiP (Jørgensen et al., 2000). In human liver cells overexpressing the wild-type and mutant receptors (W556S and C646Y), GRP78 strongly interacts with mutant LDLR whereas GRP78-wild type LDLR interaction is weak, suggesting a key role for this chaperone in ER-retention/quality control of class II LDLR mutants (Jørgensen et al., 2000). However, the overexpression of GRP78 was not capable of rescuing the mutants from ER retention. Nevertheless, abundant GRP78 reduces the processing time of newly synthesized wild type LDLR, suggesting GRP78 is critical in protein maturation of wild type LDLR. Other chaperones GRP94, ERP72 (PDIA4), and CNX have also been found to associate with class II mutants (G544V) but not with wild type LDLR (Sørensen et al., 2006). Recently we have also reported that three LDLR class II variants were found to be associated with ER chaperones: GRP78 (BiP), GRP94, the lectin chaperone CNX (Kizhakkedath et al., 2019). In cells overexpressing the G544V mutant, the ER-retention of the mutant was shown to induce ER-stress and activation of UPR as evidenced by the upregulation of mRNAs for GRP78, GRP94, ERP72, attributed to the activity of ER sensors IRE1 and PERK (Sørensen et al., 2006). Apart from its chaperoning activity, GRP94 also has a very specific role in the maturation and stability of wild-type LDLR, as it was shown to protect LDLR from PCSK-mediated degradation (Poirier et al., 2015).

Very little information is available on the ERAD components involved in the substrate recognition, retrotranslocation and degradation of LDLR mutants. Recent results from our lab indicate that the LDLR mutants interact with HRD1 and its partners SEL1L and OS9 (Kizhakkedath et al., 2019). Our results also demonstrated that proteasomal inhibition leads to stabilization of the ER-retained mutants, but had no effect on their folding. Further, inhibitors of ER mannosidase 1 also had a stabilizing effect on the mutants (Kizhakkedath et al., 2019). ER-retained variants of VLDLR, another LDLR family member, were also found to be degraded by the HRD1-SEL1L mediated proteasomal degradation (Kizhakkedath et al., 2018). Unlike VLDLR mutants, the ER retained LDLR mutants were not observed to be aggregation-prone, though overexpression of mutants caused ER stress (Kizhakkedath et al., 2019). The ERAD adaptor protein SEL1L is reported to also play an ERAD independent role in the maturation and processing of lipoprotein lipase (LPL) and hepatic lipid metabolism (Sha et al., 2014). The cell-surface rescue of an ER-retained LDLR mutant was demonstrated to be possible by the use of a chemical chaperone 4-phenylbutyrate (4-PBA) (Tveten et al., 2007). It was later revealed that 4-PBA targets COPII protein and reduces the stringency of ER-retention of misfolded substrates (Ma et al., 2017). It was suggested that stringent ER retention of misfolded substrates requires the efficient packaging of p24-family of proteins via the B site of the COPII coat and 4-PBA competes with p24 and reduces this stringency (Ma et al., 2017). The available information about the ERAD of misfolded LDLR class II mutants are limited and more detailed investigations utilizing cellular models derived from FH patients or model systems expressing physiological levels of LDLR mutants are still required to enhance our understanding of the specificities. However, it is likely that many of the ER factors involved in the folding pathway and physiological quantity control of LDLR participate in some of these processes (Figure 2).

The role of non-canonical ERAD pathways in the degradation of LDLR class II mutants have not been explored to our knowledge. We have reported previously that a small fraction of ER-retained VLDLR missense mutants are aggregation-prone and may undergo an autophagy-related process for degradation (Kizhakkedath et al., 2018). It is probable that aggregation-prone class II LDLR mutants also might undergo non-canonical ERAD.

### A Possible Role of ERAD Components Implicated in Misfolded Membrane Proteins in the ERAD of LDLR Mutants

The mechanisms by which the mutations affecting the TM domain of LDLR cause FH are only emerging. It was reported that a mutation affecting the TM domain of the LDLR (G805R), undergoes ectodomain cleavage by a metalloproteinase in the ER and results in lower LDLR levels at the cell surface (Strøm et al., 2014). The ER-resident rhomboid protease RHBDL4 is proposed as a likely candidate for this metalloproteinase. The cleaved ectodomain however does not undergo proteasomal degradation, instead, appear to pass through the secretory pathway and eventually get secreted to the extracellular space (Strøm et al., 2014). Subsequent studies revealed that many mutations affecting the TM domain of LDLR interfere with membrane-insertion of LDLR are subjected to diverse processes such as metalloproteinase cleavage, complete extracellular secretion or rapid degradation at the cell surface (Strøm et al., 2015). The underlying mechanisms of low cell-surface expression of some of these mutants were elusive (Strøm et al., 2017) and it has been proposed that the mutations affecting the TM domain of LDLR must therefore be considered to be a separate class.

## Deregulation of ER Homeostasis and Activation of UPR by LDLR Missense Mutants

Accumulation of unfolded proteins in ER activates a battery of cellular stress responses, altogether called as UPR. The UPR aims to restore the normal ER-homeostasis, however, if the stress is severe and irreversible, then UPR switches to apoptosis. Sørensen et al. (2006) for the first time reported that overexpression of LDLR mutants causes ER-stress and elicit UPR (Figure 2). Recent studies from our lab have also confirmed that LDLR mutants retained in ER results in the activation of UPR (Kizhakkedath et al., 2019). However, quite surprisingly, studies focusing on ER-stress, especially the link between ER-stress activation and cellular signaling process that modulate cell fate are missing. These studies are significantly relevant in understanding the molecular pathology of FH where liver damage due to cell death is a critical factor. The following is a detailed picture of the research that has been done pertaining to ER-stress in cell line models expressing mutant LDLR. We also briefly discuss the contradicting findings in stem cell model of FH where ER-stress is not activated when mutant LDLR is expressed.

### UPR: An Overview

Endoplasmic reticulum serves as a site for protein synthesis, folding as well as the internal cellular calcium reservoir and hence plays a critical role in cell physiology. Disruption of ER homeostasis due to derailed calcium physiology; redox imbalance; accumulation of misfolded proteins causes ER-stress (Almanza et al., 2019). Cells respond to ER-stress by activating components of counter stress response mechanisms together named UPR. UPR predominantly involves the shutdown of protein translation to reduce further protein load in ER, transcriptional upregulation of ER chaperones to assist protein folding and retrotranslocation of irreversibly misfolded proteins via ERAD. UPR is initially aimed to alleviate ER-stress and regain the normal ER-physiology. However, if the stress persists and the damage is irreversible, then the initial adaptive UPR switches to ER stress-induced apoptosis (Szegezdi et al., 2006; Almanza et al., 2019). UPR is initiated by three major ER stress sensors; PERK, IRE1, and ATF6. The three ER stress sensors are maintained inactive in resting cells by binding to ER chaperone BiP. During ER stress, BiP, which has more affinity to misfolded proteins detaches from the ER sensors and causes the activation of the latter. The concerted cellular response to ER stress is largely mediated by these sensors (Almanza et al., 2019).

Activated PERK phosphorylates the eukaryotic initiation factor 2 alpha (eIF2 alpha) (Schröder and Kaufman, 2005). Phosphorylation of eIF2 blocks cap-dependent translation and thereby reduce further protein load in the ER. Interestingly, certain mRNAs such as ATF4 which possess internal ribosome entry sites at 5′ at their untranslated regions can bypass the PERK-eIF2 alpha pathway mediated translational block. ATF4 up-regulates the expression of ER chaperones as well as induces CHOP, a pro-apoptotic transcriptional factor that induce apoptosis by repressing the anti-apoptotic protein Bcl-2. Initial PERK activation mounts a pro-survival adaptive UPR, however, persistent activation of PERK due to unresolved ER-stress leads to ATF4 mediated transcriptional induction of CHOP which switches initial adaptive UPR to ER-stress mediated apoptosis. ATF6 is cleaved by two Golgi resident proteases named site-1 and site-2 to generate an active transcriptional factor which induces the expression of ER chaperones and folding enzymes. Apart from the ER chaperones, ATF6 induces the upregulation of XBP1 mRNA, which is further processed by splicing into a smaller mRNA (XBP1s) by the ribonuclease activity of IRE-1, another ER stress sensor. Similar to ATF-6, the protein encoded by XBP1s is an active transcription factor which induces the expression of ER chaperones, folding enzymes and ERAD components (Figure 2).

Activation of UPR is aimed to resolve the stress in ER and bring back the normal ER homeostasis. Notably, major targets of all three ER stress sensors are directed to mitigate any harmful consequences of ER stress. However, when stress is overwhelming and beyond the capacity of ER adaptive stress machinery then the initial adaptive UPR switches to ER-stress activated apoptosis. Persistent activation of three major ER sensors results from unresolvable ER-stress which activates ER-apoptotic signaling principally mediated by pro-apoptotic proteins involving JNK, CHOP and BCL-2 family proteins (Szegezdi et al., 2006). ER-stress induced apoptosis has been implicated as a contributing factor in the pathophysiology of cardiovascular diseases and liver fibrosis (Maiers and Malhi, 2019), two major disease conditions manifested in FH patients.

### ER-Stress and Activation of UPR in Cell Line Models Expressing LDLR Mutants

Surprisingly, only few studies pertaining to the association of ER-stress with the molecular pathology of FH have been reported (Jørgensen et al., 2000; Sørensen et al., 2006; Kizhakkedath et al., 2019) in contrast to the fact that around 50% of LDLR mutations implicated in FH are class II mutants that are retained in ER due to misfolding. Two decades back, an interesting study by Jorgensen et al. demonstrated ERQC system play a critical role in the proteostasis of class 2 mutant LDLR proteins (Jørgensen et al., 2000). However, it was Sørensen et al. who established for the first time in 2006 that ER-retained LDLR mutants activates ER-stress (Sørensen et al., 2006). Detailed investigations on the activation of UPR by LDLR mutants revealed transcriptional induction of ER chaperones as well as the activation of three UPR sensors (Sørensen et al., 2006).

Our lab has recently reported that two missense LDLR mutants D482H and C667F associated with FH were misfolded and retained in ER (Kizhakkedath et al., 2019). Further analysis of ER-stress markers in cells expressing the aforesaid mutants pointed activation of UPR. Similar to the previous reports (Jørgensen et al., 2000; Sørensen et al., 2006), we also found ER-retained LDLR mutants strongly interact with GRP78 and other ER chaperones suggesting critical role of these chaperones in ER retention and further ERAD processing of LDLR mutants. Interestingly, our studies disclosed ER-retained LDLR mutants remained soluble in ER lumen which indicates UPR mediated induction of ER chaperones successfully chaperone the mutants and block protein aggregation. However, despite the induction of ER chaperones by UPR, mutant LDLR were not folded and transported to the cell surface. It is well known that ER stress diminish the ERAD capacity of ER and UPR mediated transcriptional induction of ER chaperones is required to sustain the ERAD machinery (Travers et al., 2000). The inferences from ER-stress studies in cell line models overexpressing LDLR mutants suggest that the activation of UPR augment ERAD process where cells can eliminate the unfolded LDLR mutants via ERAD and thereby mitigate toxic ER stress which otherwise activates cell death.

Another important aspect to consider is the role of ER-synthesized sterol regulators such as proprotein convertase subtilisin/kexin type 9 (PCSK9) in the ER physiology of class II LDLR mutant expressing cells. Both PCSK9 and LDLR are transcriptionally upregulated by SREBP2, an ER-resident transcriptional factor that binds to sterol regulatory elements in the promoter regions of sterol inducible genes including LDLR and PCSK9 (Maxwell et al., 2003). PCSK9 is expressed as a pro-form which is autocatalytically processed in ER and the active form is secreted (Seidah et al., 2003). Interestingly, PCSK9 targets surface expressing LDLR for degradation and negatively modulates the latter’s function. Decreased LDLR surface expression and increased serum LDL levels have been reported in FH patients with gain-of-function mutation in PCSK9 (Abifadel et al., 2003; Cameron et al., 2006). In contrast, the African population harboring loss-of-function PCSK9 mutants (unprocessed) have been reported to have lesser occurrence of cardiovascular diseases due to increased expression of surface LDLR and reduced serum LDL levels (Cohen et al., 2005). The aforementioned findings had also raised important questions on how cells manage the ER accumulation of unprocessed PCSK9 mutant pro forms. Does this cause ER-stress? Also, it had been quite intriguing how LDLR and PCSK9 co-exist in the same secretory pathway despite the former being a target of the latter. Hence it was widely speculated that PCSK9 would have interacting protein partners in ER. It was until 2015, Poirier et al. (2015) demonstrated that GRP94, an ER chaperone specifically interacts with PCSK9 and blocks its interaction with LDLR. Interestingly, it was later identified that GRP94 chaperones PCSK mutant pro-forms in ER and alleviate the potential toxic ER-stress (Lebeau et al., 2018). The underlying mechanism was further delineated to GRP94 interaction with mutant PCSK9 which prevents the latter’s binding to GRP78, an ER luminal chaperone that signals proteotoxic stress in ER to major ER stress sensors (Lebeau et al., 2018). In a way, contradicting to the blocker role of GRP94 in PCSK9-LDLR interactions, PCSK9 is also reported to act as a chaperone for LDLR. In ER, PCSK9 binds to LDLR and aids the transport of the latter (Stroøm et al., 2014). Interestingly, binding to LDLR augments the autocatalytic processing of PCSK9. However, it is important to note that neither the PCSK9 mutants chaperone LDLR nor the class II LDLR mutants are chaperoned by PCSK9. In fact, reduced levels of PCSK9 has been reported in FH patients harboring class II LDLR mutants (Cameron et al., 2012). It is yet to be determined how GRP94 modulates PCSK9-LDLR or PCSK9-class II mutant LDLR interactions. It is also worthwhile to investigate the efficiency of PCSK9 processing in hepatocytes homozygous for class II mutant LDLR. The other cardinal question is whether class II mutant LDLR causes PCSK9 pro-form accumulation and consequent ER-stress which further compound the already de-regulated ER homeostasis in FH patients. It remains largely unknown whether the mutual dependence of these functionally antagonizing proteins (PCSK9 and LDLR) contribute to ER physiology and cholesterol homeostasis in FH patients.

Interestingly, ER stress induced by pharmacological ER stress inducers appear to inhibit the secretion of PCSK9 due to their retention in the ER by GRP94 (Lebeau et al., 2017). This is an interesting finding since LDLR class II mutants are known to induce ER-stress and it remains to be found whether PCSK9 is retained and non-functional in this context. Chemical chaperones or pharmacological chaperones (PCs) have been identified as a promising new strategy to re-instate ER-Golgi-cell surface transport of ER-retained mutant proteins including class II LDLR mutants that retain their original biological function to some extent (a detailed review is performed in the coming section “Therapeutic Potential of Pharmacological Chaperones (PCs) and Proteostasis Regulators (PRs) in the Disease Management of FH”). Does PCSK9 remains ER-retained and non-functional when chemical chaperones are used to target LDLR mutants? The absence of functional PCSK9 might be an added advantage as it increases the number of cell surface LDLRs re-instated by the intervention of PCs. Taken together, the activation of ER stress by LDLR mutants and the inhibition of PCSK9 secretion by ER-stress activation can be well exploited for therapeutic management of FH. However, it has to be determined, to what extent ER stress is activated in cells of FH subjects, whether it is in the adaptive range where cellular UPR is equipped to manage the constant levels of ER stress or a severe irreversible ER stress where adaptive UPR response switches to apoptosis.

Also, it is quite important to note that in protein conformation diseases such as alpha-1 antitrypsin deficiency, ER-stress induced apoptosis plays a critical role in the molecular pathology associated with liver failure (Lawless et al., 2004). Adding further, ER-stress has been implicated in the pathology of various diseases such as diabetes, cystic fibrosis and neurodegenerative diseases (Almanza et al., 2019). It remains to be identified whether ER-stress play any role in the pathogenesis of class II mutants associated with FH. In order to establish a link between ER-stress and FH pathology, lymphocytes and fibroblast from FH patients expressing class two LDLR mutants have to be studied for ER-stress activation. It is known that cellular consequence to misfolded proteins retained in ER varies depending on the: mutations, tissue types, between physiological conditions of the same patient (Kim, 1998). It has to be identified whether ER-stress response to various LDLR mutations has any role in the phenotypic variation between FH patients. It is also an informed presumption that UPR activated in cells expressing mutant LDLR aids cell survival by eliminating the misfolded via ERAD. However, this has to be proved in cells from FH patients.

### ER-Stress Studies in Induced Pluripotent Stem Cells (iPSCs) Model for FH Expressing Class II Mutant LDLR

Induced Pluripotent Stem Cell (iPSCs) have been developed by reprogramming fibroblasts from a FH patient carrying a homozygous three-base pair deletion in LDLR exon 4. The mutation results in ER-retention of LDLR and hence comes under class II mutation (Omer et al., 2017). Apart from being a clinically relevant model, class II iPSCs also shows potential for stem cell-based therapy for FH. Genome editing mediated by CRISPR-Cas9 tool successfully corrected the mutation and rescued LDLR function. An interesting recent study by the same group illustrated that FH class II iPSCs and hepatocytes derived from these iPSCs elicit no ER-stress response when LDLR mutants are induced by statins (Omer et al., 2020). Statins are drugs extensively used to reduce serum LDL-cholesterol. They inhibit the enzyme HMG-CoA reductase involved in the synthesis of mevalonate from which the body makes sterols including cholesterol. Statins are known to up-regulate the expression of LDLR. Adding further, the study reveals statin mediated induction of LDLR is higher in FH class II iPSCs compared to the CRISPR corrected ones (Omer et al., 2020). However, the induced mutant LDLR which is trapped in ER elicits no ER-stress response. The report surprisingly contradicts other studies including from our lab where UPR is activated upon the expression of mutant LDLRs (Sørensen et al., 2006; Kizhakkedath et al., 2019). Although ER-stress biology of stem cells is yet to be fully understood, iPSCs are capable of activating UPR in response to pharmacological inducers of ER stress such as tunicamycin. Statins do inhibit UPR in some models, however, lipoprotein deficient serum, which is also known to induce LDLR expression, fails to activate UPR in FH class II iPSCs (Omer et al., 2020). The absence of UPR in response to mutant LDLR accumulation in ER is intriguing and one elementary clarification is that, the amount of induced LDLR mutants falls below the threshold to induce any considerable ER-stress response. It is also possible that the particular mutant used in this study (Omer et al., 2020) is only partially retained in the ER as one can infer from the data where there is a significant presence of the mature form of LDLR in response to statin treatment. The other point to be considered is the efficient removal of mutant LDLR by ERAD and therefore less likely to induce any considerable amount of ER stress. Studies involving analysis of glycosylation status and protein turn over kinetics would clarify whether the accumulated LDLR is completely or partially retained in ER. In cell line overexpressing models, LDLR mutants are expressed from CMV promoters and each cell carries more than one copy of the plasmid and consequently, the protein is expressed in enough quantity to mount an ER-stress response. A comparative protein expression study involving cell line overexpression models and FH class II iPSCs is required to ascertain the above-said assumption.

## Therapeutic Potential of Pharmacological Chaperones (PCs) and Proteostasis Regulators (PRs) in the Disease Management of FH

Research underpinning the molecular pathology of FH from various labs including our group have demonstrated that class II mutations in LDLR cause misfolding, ER retention and consequent protein degradation via ERAD (Sørensen et al., 2006; Kizhakkedath et al., 2019). As abundantly mentioned in this review, ER protein quality control systems (ERQC) maintain proteostasis by facilitating protein folding and eliminates misfolded proteins via ERAD. Sophisticated ERQC comprises ER chaperones as well as the protein components of UPR, ERAD and cytosolic proteasomal degradation machinery. Decades-long research in ERQCs led to the development of various proteostasis regulators (PRs) and PCs that either positively or negatively modulates ERQC components (Gámez et al., 2018). Currently, the first-line therapy for FH include statins which either exert their lipid-lowering effect through the inhibition of HMG-CoA reductase or via SREBP activation, which in turn induces LDLR expression. However, SREBP2 also induces the expression of PCSK9, which targets LDLR for lysosomal degradation (Dubuc et al., 2004). Therefore, novel targets for the modulation of LDLR expression and function are increasingly being sought as a supplementation therapy with statins.

Multiple reports are available evidencing the successful application of PCs and PRs in various protein conformation diseases (Mu et al., 2008; Mohamed et al., 2017; Gámez et al., 2018). This suggests that PCs and PRs are promising candidates in the clinical management of FH. It is interesting to note that the idea of using PCs or PRs emerged from an early observation where ΔF508 CFTR, a single phenylalanine deletion mutant found in more than 85% of cystic fibrosis alleles was functional when expressed in *Xenopus* oocyte grown at room temperature (Drumm et al., 1991). Normally, ΔF508 CFTR is misfolded and retained in ER followed by degradation via ERAD. The functional correction of ΔF508 CFTR at low temperature was later established *in vitro* in cell lines (Denning et al., 1992). This suggests that restoration of protein transport and functionality of ER-retained mutants can be achieved by modulating proteostasis.

Proteostasis regulators are very often small molecule modulators of protein homeostasis (Balch et al., 2008). PRs predominantly act by manipulating the cellular stress response pathways including UPR. PRs have been proved to rescue misfolded proteins from ER retention either by modulating ERAD or by enhancing the expression of ER/cytosolic chaperones. PRs such as celastrol, curcumin, and HSP90 inhibitors induce the expression of cytosolic chaperones, Kifunensine and Eeyarestatin I inhibit ERAD, thapsigargin modulates calcium signaling and activates UPR (Wang et al., 2011; Gámez et al., 2018).

Unlike PRs, PCs by itself act on the target misfolded proteins and tilt the equilibrium toward the folding state (Ringe and Petsko, 2009). Interestingly, PCs that have been successfully developed for misfolded enzymes are their substrate variants. Substrate binding sites or active binding sites of enzymes are generally formed by more than one domain and therefore PCs bind and aid interaction between protein domains and thereby assist correct folding (Ringe and Petsko, 2009; Gámez et al., 2018). Natural co-factors and ligands are also being used as PCs for various protein conformation diseases. PCs are often protein-specific and sometimes mutation specific. However, cases have been reported where the same PCs are found effective for various mutants of the same protein (Conn and Janovick, 2009). Adding further, the combined application of PRs and PCs can be more effective as PRs increases the cellular chaperonic capability/QCS whereas PCs increases the availability of active folded missense proteins (Gámez et al., 2018).

### Potential of PRs and PCs in Treating FH

The 4-phenyl butyric acid (4-PBA), a low molecular weight bipolar fatty acid derivative appears to rescue ER-retained transport defective class II mutant LDLR G544V in cell line overexpressing model. Although, 4-PBA mediates the rescue of only 30% of mutant LDLR expressed, the rescued LDLR mutant is expressed on the cell surface and capable of LDL binding and internalization compared to wild type (Tveten et al., 2007). Interestingly, the rescue effect is mutation-specific as other class II LDLR mutants are not rescued by 4-PBA. Recently, it has been demonstrated that 4-PBA act on the COPII machinery to promote the ER exit of the G544V mutant (Ma et al., 2017). 4-PBA has also been shown to mitigate ER-stress in animal models of neurodegenerative disorders and type 2 diabetes (Özcan et al., 2006; Bondulich et al., 2016). However, despite the promising outcome from the 4-PBA studies, no further studies have been done at preclinical level. It is also notable that very limited studies have been performed regarding the potential of established PRs and PCs in functionally rescuing the class II LDLR mutants.

Our lab has been studying proteostasis regulation of various missense mutants implicated in various genetic diseases including FH. We have demonstrated that the genetic ablation of ERAD components leads to the stabilization of ER-retained VLDLR missense mutants (Kizhakkedath et al., 2018). However, we are yet to demonstrate whether the rescued mutants are functional. Pharmacological inhibitors of ERAD such as Kifunensine and Eeyarestatin I are found to functionally rescue missense mutants associated with lysosomal storage diseases (Wang et al., 2011). Kifunensine inhibits Mannosidase which is a critical component in the recognition of misfolded proteins that are marked for ERAD. Eeyarestatin I blocks the extraction of ubiquitinated proteins from the ER membrane by inhibiting p97 ATPase activity (Wang et al., 2011). Even though we have demonstrated the stabilization of LDLR missense mutants in cell lines (Kizhakkedath et al., 2019), it is yet to be studied whether the aforementioned small molecule ERAD inhibitors have any impacts on the functional rescue of LDLR mutants.

The UPS is the end component of ERAD where the retrotranslocated misfolded proteins are finally processed. We and others have shown that proteasome inhibitors such as MG132 aid protein transport and significantly improve the protein function of missense mutants which otherwise retained in ER and subsequently subjected to ERAD (Wilke et al., 2012; Kizhakkedath et al., 2018). It is quite intriguing how inhibition of proteasome rescues misfolded proteins from ER retention and subsequent ERAD. As reported earlier (Pirkkala et al., 2000), inhibition of proteasome causes perturbation in proteostasis which elicit proteotoxic stress response with transcriptional induction of chaperones. The increase in chaperone reservoir aid protein folding and thus probably explains the partial rescue of ER-retained misfolded proteins and consequent transport to its destined locations. From our studies and other published reports, one can presume that partial functional rescue of ER retained mutant proteins by ERAD blockers and proteasome inhibitors are either mediated by the increased expression of chaperones due to perturbance in proteostasis or the increased boarding of misfolded proteins in ER due to the blocked ERAD. The increased duration of misfolded proteins in the ER lumen might increase their chances to get transported out to Golgi. It would be interesting to investigate these aspects. The exact mechanism by which the inhibition of ERAD or proteasome leads to functional rescue of misfolded proteins is yet to be studied in depth. Proteasome inhibitors such MG132 acts on multiple pathways, the synergistic effect of which has been shown to be protective during acute myocardial ischemia (Yu and Kem, 2010). The cardioprotective mechanisms mediated by MG132 were proposed to be through degradation of IkB (inhibitory kB), GRK-2 (G-protein-receptor kinase 2), ARC (apoptosis repressor with caspase recruitment domain), and also by induction of HSP (Yu and Kem, 2010). In HepG2 cells, other than proteasomal inhibition, MG132 has been shown to enhance LDL uptake by upregulating LDLR mRNA expression through a PKC-dependent pathway. An unexpected effect of MG132 was the suppression of PCSK9 expression, which aided in decreased LDLR degradation and enhanced LDL uptake (Yan et al., 2014). Curcumin is a plant-derived natural polyphenolic compound which has been demonstrated to induce HSPs and reported to have anti-inflammatory, antioxidant properties, in addition to preventing protein-aggregation (Maiti et al., 2014). Curcumin has been recently reported to produce a hypocholesterolemic effect by enhancing the cell-surface expression of LDLR and LDL uptake through downregulation of PCSK9 gene expression in HepG2 cells (Tai et al., 2014). The effect of curcumin on the rescue of mutant LDLR misfolding has not been investigated so far and can be explored. As mentioned above, only a few studies have been performed on the potential roles of PCs and PRs in the rescue of class II LDLR mutants. More investigations involving already established PRs and PCs that show rescue potential in other protein conformation diseases might improve the chances of finding effective PCs/PRs for FH class II mutant LDLRs.

### Potential Challenges in Using PRs and PCs as Drugs for FH

Even though, the cell line-based studies are encouraging, targeting normal cellular process such as ERAD and proteasome has deleterious effects. One has to be cautious of the fact that majority of the information on the molecular pathology of class II LDLR mutants come from cell line-based overexpression studies. The cell line-based data has to be validated with studies involving tissues from FH patients. Low efficiency also poses a problem while considering PCs/PRs as therapeutic agents. Cell line-based study shows only 30% functional recovery of rescued LDLR by 4-PBA, an extensively studied chemical chaperone for various mutants associated with protein conformational diseases. Pre-clinical studies show that application of PCs result in variable increase in the activity of mutant enzymes associated with lysosomal storage diseases (Parenti et al., 2015). Some mutations respond well and others not at all (Parenti et al., 2015). Hence, the efficiency of PCs, in general, is debatable. Therefore, it is a long way ahead to determine whether restoration of mutant LDLR activity by 4-PBA is beneficial to FH patients.

It is quite interesting to note that 4-PBA mediated rescue of LDLR mutants is mutation specific. The authors diligently prove that the other mutations in the same domain of the protein which are also class II mutations are not rescued by 4-PBA (Tveten et al., 2007). One can reasonably assume that mutations that grossly affect the protein stability or core LDL binding site may not be rescued by PCs. However, regardless of various limitations, PCs and PRs have the potential to be considered as therapeutic agents for FH patients with class II LDLR mutations.

## Conclusion and Future Perspectives

In this review, we have outlined the impact of class II LDLR mutants on ER-proteostasis and how it can be modulated for the therapeutic management of FH. Conventional lipid-lowering drugs are effective in maintaining LDL-C levels in heterozygous FH patients since these patients have residual LDLR function due to the presence of a normal copy of the *LDLR* gene. In homozygous FH patients where a functional LDLR is lacking, the current LDL-C lowering drugs have minimal effect (Wilemon et al., 2020). A growing body of evidence suggests that ER-proteostasis can be modulated for therapeutic purposes to treat various protein conformation diseases including class II FH. LDLR is the founding member of the LDL receptor family whose members share structural and functional domains. The exquisite structure of these class of proteins requires a specialized array of private and global quality control factors, many of which remain to be unraveled. Despite the significant development in ERAD research, only limited information is available on the factors responsible for the recognition, ER-retention and degradation of defective LDLRs. Notably, we are yet to find out whether non-canonical ERAD pathways such as ER-phagy and microautophagy are involved in the disposal of misfolded mutant LDLRs. Cell line models of class II mutants associated with FH indicate a role for ER-stress and UPR activation in the pathogenesis. More studies using fibroblasts from heterozygous and homozygous FH patients with diverse Class II LDLR mutations will clarify this aspect and lay foundations for designing therapies focused on adaptive UPR and folding-rescue of these class of mutants. A growing body of evidence suggests that PCs and PRs have the potential to augment conventional therapies for FH. Though the benefits have to be carefully weighed against manipulating the natural processes of ERAD and proteostasis, PRs have the potential to be considered for therapeutic management of FH. Cholesterol is a constituent of the ER membrane and is regulated by ER. Therefore, mechanisms aimed at restoring ER homeostasis are likely to influence cholesterol homeostasis (Fu et al., 2012). Additional investigations on the influence of PRs on cholesterol metabolism independent of proteostasis would also provide insights into novel pathways of LDLR regulation.

## Author Contributions

DO, PK, and AJ drafted different sections of the manuscript. PK and BA compiled and edited the manuscript. PK and DV created the figures. DO, PK, DV, AJ, and BA critically reviewed and edited the manuscript. All authors contributed to the article and approved the submitted version.

## Conflict of Interest

The authors declare that the research was conducted in the absence of any commercial or financial relationships that could be construed as a potential conflict of interest.

## Abbreviations

CAD: coronary artery disease
CMV: cytomegalovirus
ERAD: endoplasmic reticulum associated degradation
ER: endoplasmic reticulum
FH: familial hypercholesterolemia
LDLR: low-density lipoprotein receptor
UPR: unfolded protein response.
