# Supplementary material for: Proteostasis Regulation in the Endoplasmic Reticulum: An Emerging Theme in the Molecular Pathology and Therapeutic Management of Familial Hypercholesterolemia
Source: Front Genet. 2020 Sep 23;11:570355. doi: 10.3389/fgene.2020.570355 (PMC7538668; doi:10.3389/fgene.2020.570355)
Supplement: Supplementary file 1 [file Data_Sheet_1.PDF]

# Supplementary Figure S1

## A. All reported missense mutations in LDLR

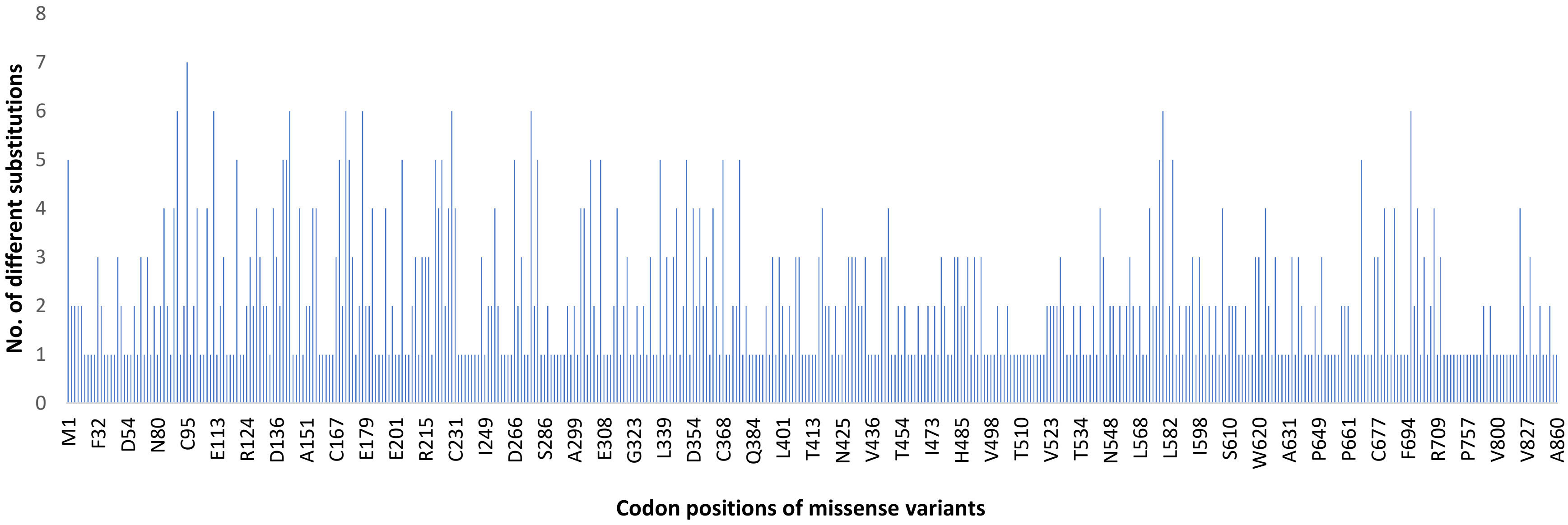


## B. Corresponding amino acid positions of different LDLR domains

| LDLR domains | LDL-receptor class A | LDL-receptor class A | LDL-receptor class A | LDL-receptor class A | LDL-receptor class A | LDL-receptor class A | LDL-receptor class A | EGF-like 1 | EGF-like 2 | LDL-receptor class B 1 | LDL-receptor class B 2 | LDL-receptor class B 3 | LDL-receptor class B 4 | LDL-receptor class B 5 | LDL-receptor class B 6 | EGF-like 3 | O-Linked sugar domain | Transmembrane domain | Cytoplasmic domain |
|---|---|---|---|---|---|---|---|---|---|---|---|---|---|---|---|---|---|---|---|
| Position(s) | 25- 65 | 66- 106 | 107- 145 | 146- 186 | 195- 233 | 234- 272 | 274- 313 | 314- 353 | 354- 393 | 397- 438 | 439- 485 | 486- 528 | 529- 572 | 573- 615 | 616- 658 | 663- 712 | 721- 768 | 789- 810 | 811- 860 |
| Length | 41 | 41 | 39 | 41 | 39 | 39 | 40 | 40 | 40 | 42 | 47 | 43 | 44 | 43 | 43 | 50 | 48 | 22 | 50 |

**Figure S1A**: Codon positions of all reported missense mutations in HGMD (n=451) and the number of times different substitutions have been reported. **Figure S1B**. The positions of codons contributing to each LDLR domains and the length of each domain are provided for comparing the distribution of mutations across each domain.
